# CD40–CD40L in Neurological Disease

**DOI:** 10.3390/ijms23084115

**Published:** 2022-04-08

**Authors:** Heather D. Ots, Jovanna A. Tracz, Katherine E. Vinokuroff, Alberto E. Musto

**Affiliations:** 1 Department of Pathology and Anatomy, Eastern Virginia Medical School, Norfolk, VA 23510, USA; otshd@evms.edu (H.D.O.); traczja@evms.edu (J.A.T.); vinokuke@evms.edu (K.E.V.); 2 Department of Neurology, Eastern Virginia Medical School, Norfolk, VA 23510, USA

**Keywords:** neuroinflammation, CD40, CD40 ligand, immunotherapy

## Abstract

Immune-inflammatory conditions in the central nervous system (CNS) rely on molecular and cellular interactions which are homeostatically maintained to protect neural tissue from harm. The CD40–CD40L interaction upregulates key proinflammatory molecules, a function best understood in the context of infection, during which B-cells are activated via CD40 signaling to produce antibodies. However, the role of CD40 in neurological disease of non-infectious etiology is unclear. We review the role of CD40–CD40L in traumatic brain injury, Alzheimer’s Disease, Parkinson’s Disease, stroke, epilepsy, nerve injury, multiple sclerosis, ALS, myasthenia gravis and brain tumors. We also highlight therapeutic advancements targeting the CD40 system to either attenuate the neuroinflammatory response or leverage the downstream effects of CD40 signaling for direct tumor cell lysis.

## 1. Introduction

Neuroinflammatory responses are mediated by neurons, astrocytes, microglia, and endothelial cells through a complex signaling network of cytokines, chemokines, and secondary messengers that alter the chemical composition of the neural microenvironment [1]. Whereas this neuroinflammatory cascade is critical when coordinating the body’s physiological response to external and internal noxa, unresolved neuroinflammation can promote chronic disease [2]. 

The CD40 receptor and CD40L ligand are transmembrane proteins that belong to the tumor necrosis factor (TNF) receptor superfamily and are critical to the initiation and sustainment of the inflammatory response [3]. The CD40 dyad was first identified for its role in B-cell activation for antibody production and proliferation of inflammatory cells such as macrophages and lymphocytes in response to infection. However, its role in neurological disease of non-infectious etiology has recently gained the attention of neuroscientists and neurooncologists, as the aberrant expression of CD40 can be either (1) detrimental to the survival of neural tissue, for example, in autoimmune neurological disorders such as multiple sclerosis, or (2) beneficial in activating the immune cells necessary for tumor cell lysis. We review the effects of CD40 signaling in traumatic brain injury, Alzheimer’s Disease, Parkinson’s Disease, stroke, epilepsy, nerve injury, multiple sclerosis, amyotrophic lateral sclerosis, myasthenia gravis, and brain tumors, and highlight recent therapeutic advancements targeting the CD40–CD40L system.

## 2. CD40–CD40L Molecular Signaling Overview

**Figure 1:**
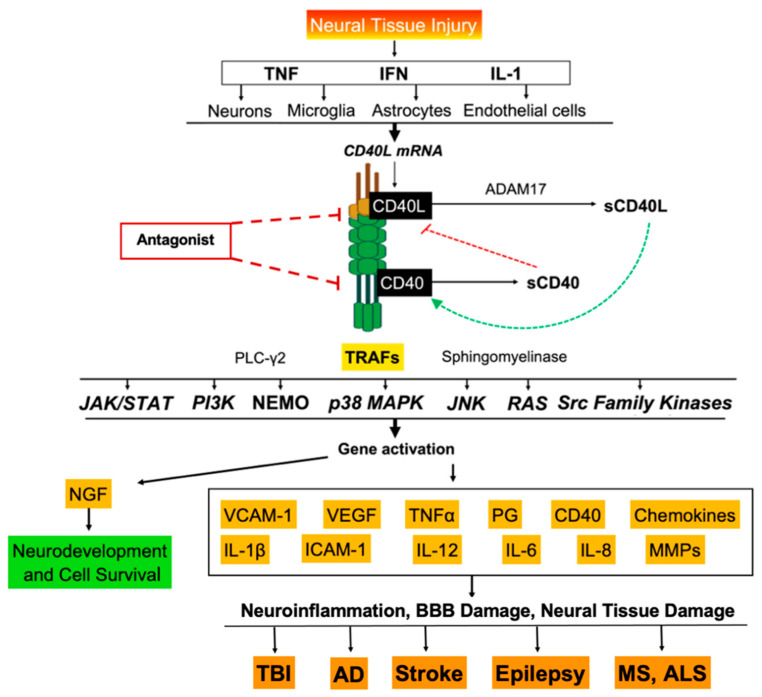
Effects of CD40–CD40L ligation in neurology. Proposed downstream expression of cytokines, chemokines, and cell-adhesion molecules following CD40–CD40L activation that contribute to neuroinflammation and damage to the blood–brain barrier (BBB) and neural tissue in traumatic brain injury (TBI), Alzheimer’s disease (AD), stroke, epilepsy, multiple sclerosis (MS) and amyotrophic lateral sclerosis (ALS). Upon CD40–CD40L ligation, soluble CD40 is released, which can bind to membrane CD40L to inhibit further CD40–CD40L-mediated immune responses.

The CD40 receptor (TNFRSF5) is 48-kDa type 1 transmembrane protein [3]. Although multiple isoforms of the CD40 receptor exist, two predominate in humans: the signal-transducible CD40 type I receptor and a C-terminal truncated non-signal-transducible CD40 type II receptor [4]. The CD40 ligand (CD40L, CD154) is a 32–39 kDa type II transmembrane protein [3]. In response to infection, both CD40 and CD40L are presented on the surface of B and T-cells respectively, which, upon ligation, activates B-cells for antibody isotype switching and upregulates the production of pro-inflammatory cytokines in attempt to neutralize the pathogen. The process of signal transduction includes the recruitment of TNF Receptor-Associated Factors (TRAFs) which function as adaptor proteins to initiate intracellular signaling cascades such as the phosphatidylinositol 3-kinase/Akt (PI3K), p38 mitogen-activated protein kinase (p38 MAPK), NF-κB essential modulator (NEMO), Jun N-terminal kinase (JNK), Ras, and Src Family Kinase (Src) pathways (Figure 1) [3,5,6]. The production and release of pro-inflammatory cytokines, angiogenic factors, prostaglandins, cellular adhesion molecules and chemokines including IL-1, TNFα, IL-8, VEGF, ICAM-1, and VCAM-1 follows shortly thereafter (Figure 1) [7,8,9].

CD40 and CD40L are also found in soluble forms, sCD40 and sCD40L respectively, which are synthesized via cleavage of the extracellular domain from their membrane-bound counterparts (Figure 1) [10,11]. sCD40L release—facilitated by the proteolytic enzymes ADAM10 and ADAM17—occurs upon CD40 ligation, and thus is used as a biomarker of CD40-mediated inflammatory activity [11]. Whereas sCD40L binds the CD40 receptor to initiate proinflammatory signaling, sCD40 has been shown to antagonize the CD40–CD40L interaction, as it can bind membrane CD40L to either reduce or prevent further signaling, suggesting an autocrine regulatory role [12,13]. 

CD40–CD40L signaling is upregulated by (1) the JAK/STAT pathway (2) TNFα through NF-κB and SMAR1, (3) IFN-γ through STAT1, and (4) IL-1β [14,15].

## 3. CD40–CD40L in Neurological Diseases

**Figure 2:**
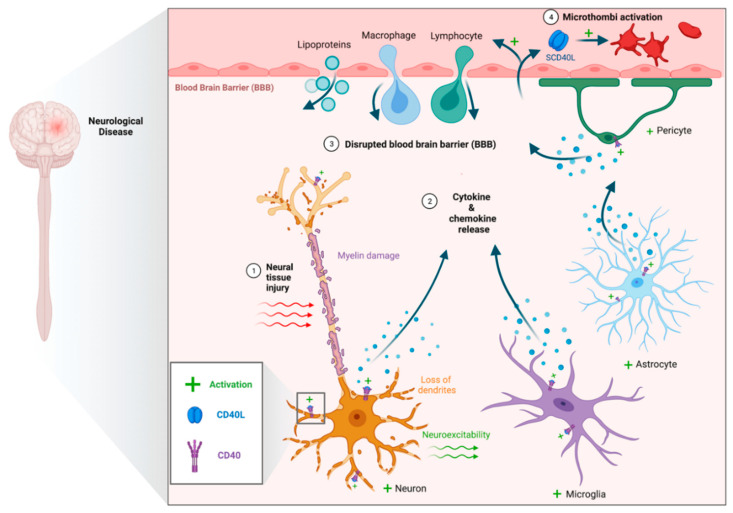
CD40–CD40L in neuroinflammation. Proposed mechanism for cytokine-mediated neuronal damage, disruption of the blood brain barrier, and microthrombi formation.

**Figure 3:**
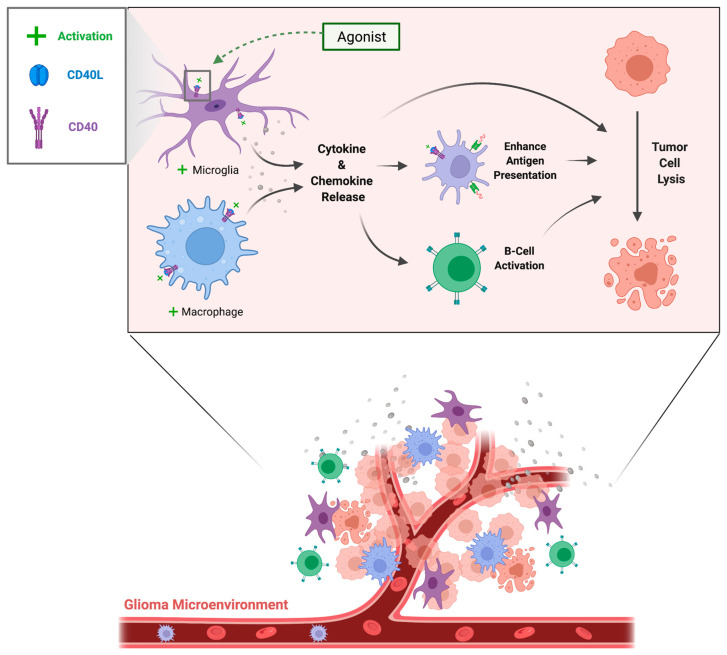
CD40–CD40L activation promoting glioblastoma (GBM) cell lysis. GBM is unique from other neurological diseases of noninfectious etiology in that CD40 signaling is downregulated rather than amplified by tumor cells as they evade the immune response.

While the majority of neurological diseases described below have several factors contributing to pathogenesis, aberrant neuroinflammation mediated by CD40–CD40L increases BBB permeability, exacerbates edema, neuronal, and glial cell damage, and promotes the formation of occlusive microthrombi (Figure 2). Brain tumors, such as glioblastoma multiforme, present a unique role of CD40–CD40L, as activation can promote tumor cell lysis (Figure 3). 

### 3.1. Traumatic Brain Injury

Traumatic brain injury (TBI), although caused by a single event, is considered a disease process in which post-traumatic edema and increased intracranial pressure are associated with poor neurological outcomes [16]. CD40 and CD40L are expressed on activated macrophages and microglia localized to the site of the traumatic impact in animal models of TBI [17]. Further, sCD40L is considered a biomarker for poor prognosis in patients with TBI, as there exists a positive correlation between serum sCD40L levels and (1) TBI severity (as assessed by APACHE-II and GCS scores) and (2) 30-day mortality [18,19,20]. CD40-mediated neuroinflammation post-TBI may result from the formation of neurovascular thromboses with subsequent tissue ischemia [20]. In animal models, reduced expression of CD40 is associated with reduced brain edema in the 30 days following TBI [21]. This presents an opportunity to explore the effects of CD40 modulation on post-TBI edema and mortality.

### 3.2. Aging and Alzheimer’s Disease

The CD40–CD40L interaction is associated with both the developmental and neurodegenerative aspects of aging [22].

CD40 signaling promotes neurite organization, survival, and growth of axons in sympathetic neurons during the perinatal period via nerve growth factor (NGF) [23]. This pro-neurogenic function seems to reverse in late adulthood and aging, where CD40L is positively correlated with disease [24].

Alzheimer’s disease (AD), the most common neurodegenerative disease in the United States, has a significant inflammatory component, largely resulting from microglia and astrocytes activated via CD40–CD40L signaling [22,24]. CD40L-stimulation of microglia disturbs the expression of genes regulating amyloid precursor protein (APP) processing and tau phosphorylation; contributing to the formation of neurofibrillary tangles and β-amyloid plaques (characteristic accumulates in AD pathogenesis) [22]. Intense CD40L immunoreactivity occurs within astrocytes in gray matter surrounding Aβ_1–42_ plaques [22,24,25]. Thus, CD40 activation may contribute to not only the development of neurofibrillary tangles and β-amyloid plaques, but also to the inflammatory damage in the neural tissue that surrounds them.

### 3.3. Parkinson’s Disease

Current therapies available for the treatment of Parkinson’s Disease (PD) have focused on improving patient cognitive and motor function; however, they do not inherently alter the neurodegenerative processes underlying PD, such as neuroinflammation [26].

When microglia and astrocytes are stimulated via CD40 signaling, inducible nitric oxide synthase and cyclooxygenase-2—two molecules known to contribute to the pathogenesis of PD—are upregulated and cause the selective loss of dopaminergic neurons in cell cultures [27]. This evidence suggests that CD40-mediated neuroinflammation may promote the loss of dopaminergic neurons and stunt dendrite growth in PD.

### 3.4. Ischemic Stroke

CD40 signaling plays a significant role in both the predisposing neuroinflammatory etiology and the effects of ischemic stroke—the largest neurological contributor to global burden of disease [28,29,30]. CD40L is expressed by multiple cells that participate in atherogenesis [28] and is co-expressed with CD40 on vascular endothelial cells, smooth muscle cells, and macrophages in human atherosclerotic lesions in situ [30]. Additionally, CD40L has been shown to reduce the stability of atherosclerotic plaques [28].

Therapies for stroke are beginning to focus on targeting the ischemic penumbra, an area that is still salvageable if neuroinflammation is reduced and reperfusion is established [31]. As infarct volume is reduced in CD40- and CD40L-deficient mice, there exists an opportunity to explore the inhibition of CD40 signaling to salvage the penumbra [31,32].

### 3.5. Epilepsy

Neuroinflammatory pathways, activated in pharmaco-resistant epilepsy, which ~30% of epileptic patients suffer from, contribute to both the development of epilepsy and the maintenance of a chronic epileptic state [33].

In patients with epilepsy following a stroke, plasma sCD40L levels and CD40 expression in leukocytes were significantly elevated [34]. In addition, downregulation of CD40–CD40L attenuated both seizure susceptibility and severity in animal models of epilepsy [35]. This suggests that CD40-mediated inflammation could contribute to the enhanced neuroexcitability that triggers epilepsy and encourages the exploration of CD40 antagonists in future clinical trials for the treatment of epilepsy [35]. 

### 3.6. Central and Peripheral Nerve Injury

In animal models of spinal injury, there is an increase in CD40+ microglia in the dorsal horn, which promotes the infiltration of CD40L+ T-cells and perpetuates cytokine-mediated damage [36,37]. When this pathway was explored in a murine study of nerve allograph rejection, blocking CD40 mediated inflammation via administration of anti-CD40L antibodies resulted in immunologic graft tolerance [38].

Further, CD40-mediated enhancement of both calcitonin gene-related peptide (CGRP) expression in peripheral ganglia and chemokine ligand 2 (CCL2) production in the spinal cord contributes to neuropathic pain [37,39]. CD40 also contributes to microangiopathy in diabetic nerve pathology through the production of hypoxia-inducible factor-α (HIF-α) [40]. 

### 3.7. Multiple Sclerosis

Demyelinated plaques in multiple sclerosis (MS) contain inflammatory infiltrates predominantly composed of T-cells and microglia expressing CD40L and CD40 respectively [29]. When CD40L-expressing T-cells infiltrate the CNS and activate CD40 receptors on microglia, cytokines, nitric oxide, and matrix metalloproteinases are released by microglia and increase demyelination [41]. CD40 expressing B-cells have also been identified within the inflammatory lesions of deceased MS patients, suggesting that the production of antibodies through CD40-mediated T- and B-cell interactions could contribute to MS pathology [42]. Further, CD40 stimulated Th1-cells may contribute to direct myelin lysis via activation cytotoxic T-cells [42,43]. Higher numbers of peripheral CD40L+ T-cells and CD40+ dendritic cells, along with elevated cerebral spinal fluid concentrations of sCD40L were also found in patients with MS [41,44,45].

Disruption of the blood–brain barrier (BBB) in MS permits the recruitment of inflammatory cells into the brain, which then significantly disrupt myelinated axons. Inflammatory lesions resulting from MS were shown to be accompanied by CD40-mediated disturbance of the blood–brain barrier [41,44]. Crosstalk between toll-like receptor-4 (TLR4) and CD40 signaling also has a role in regulating IL-10 production by B-cells during MS relapses, suggesting CD40 may promote recovery from MS relapse if signaling occurs in parallel with TLR4 [46].

Lastly, attempts have been made to counteract dysregulated CD40 signaling in MS: defective regulation of CD40-stimulation on brain-derived neurotrophic factor levels in untreated relapse-remitting MS was found to be reversible with IFN-beta1a therapy [47].

### 3.8. Amyotrophic Lateral Sclerosis

CD40 signaling between antigen presenting cells (APCs) and T-cells is upregulated in the blood of 56% of patients with amyotrophic lateral sclerosis (ALS); a discovery that inspired the development of a monoclonal antibody to CD40L, which delayed the onset of paralysis and extended survival in murine models [48]. 

### 3.9. Myasthenia Gravis

CD40 signaling is necessary for B-cell activation and antigen-specific antibody production inherent to the development of myasthenia gravis (MG)—the most common disorder of the neuromuscular junction [49,50]. CD40L knockout mice were found to be completely resistant to MG induction [51].

Dysregulated CD40 signaling compromises immune tolerance by allowing auto-reactive T-cells to avoid negative selection; and overexpression of CD40 leads to the production of pro-inflammatory cytokines that activate such autoreactive T-cells [52]. These T-cells then go on to promote autoantibody production by B-cells, perpetuating the disease [51,53]. 

### 3.10. Brain Tumors

The CD40/CD40L axis is under investigation for its role in the progression and treatment of both primary and secondary brain tumors including gliomas [54] (Figure 3, Table 1). 

Increased CD40/CD40L expression in gliomas is associated with good prognoses [55] and CD40 agonism enhances intratumoral T-cell responses in glioma patients [54,56]. However, other studies measuring expression of CD40 in grades II, III, and IV gliomas established a negative correlation between CD40 expression and patient survival [57]. CD40 signaling also results in (1) enhanced glioblastoma multiforme (GBM) invasiveness, clonogenicity, and temozolomide resistance [58], and (2) the production of angiogenic factors (e.g., vascular endothelial growth factor [VEGF]) that promote tumor growth via neovascularization [59]. Thus, researchers are investigating combination therapies to both promote the anti-tumor effects and inhibit pro-tumor effects of CD40 signaling (Section 4) [54]. 

**Table 1:** Therapies targeting the CD40 axis with potential applications for the treatment of neurological disease (Clinical Trials, 2011—Present).

Target	Therapy	Format	Disease	Phase	Date of Trial Completion
CD40 AGONIST	Mitazalimab [60,61,62]	Anti-CD40 mAb	PDAC [60], solid tumors [61,62]	Ib/II	8/2025 [60], 2/2023 [61], 3/2017 [62]
	RO7300490 [63]	FAP-α targeted CD40 agonist	Solid tumors	I	8/2026
	CD40.HIVRI.Env Vaccine [64]	Anti-CD40 mAb fused to HIV-1 envelope protein	HIV prevention	I	12/2023
	LVGN7409 [65]	Anti-CD40 mAb	Metastasis	I	4/2023
	CDX-1140 [66,67,68,69,70]	Anti-CD40 mAb	Solid and hematological malignancies [66,67,68,69,70]	I	5/2025 [66], 12/2024 [67], 8/2023 [68], 7/2023 [69], 11/2021 [70]
	2141-V11 [71,72]	Anti-CD40 mAb	Cancer lesions to the skin [71], malignant glioma [72]	I	7/2025 [71], 12/2025 [72]
	Sotigalimab [73,74,75,76,77,78,79,80,81,82,83,84]	Anti-CD40 mAb	Melanoma [73,74,76,78,82,83,84], RCC [73,74], sarcoma [75], NSCLC [74,82,84], adenocarcinoma [77,81], PDAC [79], CNS tumors [80]	I/II	2/2025 [73], 10/2024 [74], 12/2023 [75], 12/2022 [76], 11/2022 [77,78], 9/2022 [79,80], 12/2021 [81], 11/2020 [82], 8/2020 [83], 6/2018 [84]
	Selicrelumab [85,86,87,88,89]	Anti-CD40 mAb	BCL [85], solid tumors [86,87,89], PDAC [88]	I	04/2021 [85], 10/2019 [86], 11/2019 [87], 11/2018 [88], 4/2018 [89]
	NG-350A [90]	Adenovirus expressing anti-CD40 antibody	Epithelial cancers	I	12/2021
	SEA-CD40 [91]	Anti-CD40 mAb derived from dacetuzumab	Solid tumors, lymphomas, PDAC	I	2/2024
	CP-870,893 [92,93,94,95,96]	Anti-CD40 mAb	Melanoma [92,93], mesothelioma [94], PDAC [95,96]	I	5/2016 [92], 9/2015 [93], 1/2014 [94], 4/2013 [95], 1/2011 [96]
	Chi Lob 7/4 [97]	Anti-CD40 mAb	Advanced malignancies	I	10/2014
CD40L AGONIST	AdcuCD40L [98]	Adenovirus encoding CD40L	Esophageal Carcinoma	II	7/2011
	CMN-001 [99,100,101,102,103,104,105,106,107]	Dendritic cells with RNA from tumor specimen and CD40L RNA	RCC [99,100,101,103,105,106,107], NSCLC [38], genitourinary cancer [103,104]	II/III	3/2022 [99], 5/2018 [100], 4/2018 [101], 3/2018 [102], 9/2017 [103,104], 3/2017 [105], 5/2012 [106], 2/2012 [107]
	Ad-sig-hMUC-1/ecdCD40L vector vaccine [108]	Adenoviral vector encoding hMUC-1 and CD40L	Epithelial cancer of the lung, breast, ovary, prostate, colon	I	6/2017
	AdCD40L [109]	Adenoviral vector encoding CD40L	Solid tumors	II	1/2016
	B-CLL vaccine [110,111]	Tumor cells expressing CD40L, IL-2	B-CLL	I	4/2015 [110], 8/2013 [111]
	rAd.CD40L [112]	Adenoviral vector encoding CD40L	Metastatic melanoma	I/II	3/2022
	GM.CD40L [113,114,115,116]	Irradiated tumor cells transduced with GM-CSF and CD40L	Mantle cell lymphoma [113], adenocarcinoma of the lung [114,116], MDS [115]	II	7/2021 [113], 7/2020 [114], 12/2019 [115], 2/2019 [116]
	LOAd703 [117,118,119,120]	Adenovirus encoding TMZ-CD40L and 4-1BBL	Melanoma [117], CRC [118,119], PDAC [120], ovarian cancer [120], biliary carcinoma [120]	I/II	6/2024 [117], 10/2023 [118], 12/2022 [119], 12/2021 [120]
	SL-172154 [121,122]	Fusion protein SIRPα -Fc-CD40L	Ovarian cancer [121], SCC [122]	I	7/2022 [121,122]
CD40 ANTAGONIST	CFZ533 (iscalimab) [123,124,125,126,127,128,129,130,131,132]	CD40 mAb	Kidney/liver transplant [123,124,130,132], SLE [125], SS [126,128], LN [127], MG [129], graves’ disease [131], RA [132]	II	3/2027 [123], 1/2027 [124], 10/2024 [125], 2/2024 [125], 2/2023 [126], 9/2022 [127], 6/2018 [128], 12/2017 [129], 11/2017 [130], 4/2017 [131], 2/2017 [132]
	BI 655064 [133,134,135,136,137,138,139]	CD40 mAb	LN [133,134], ITP [136], RA [137]	II	8/2021 [133], 8/2020 [134], 5/2016 [135], 4/2016 [136], 4/2015 [137], 5/2014 [138], 9/2012 [139]
	FFP104 [140,141]	CD40 mAb	PBC [140], CD [141]	II	12/2017 [140,141]
	Lucatumumab [142,143]	CD40 mAb	Lymphoma	I/II	2/2013 [142], 5/2012 [143]
	Bleselumab [144,145,146,147,148]	CD40 mAb	Kidney transplant [144,145,148], psoriasis [147]	II	10/2021 [144], 1/2017 [145], 1/2015 [146], 9/2012 [147], 1/2012 [148]
CD40L ANTAGONIST	SAR441344 [149,150]	CD40L mAb	Relapsing MS [149], SS [150]	II	1/2023 [149], 10/2022 [150]
	AT-1501 [151,152]	CD40L mAb	T1DM patients undergoing islet cell transplantation [151], ALS [152]	II	6/2026 [151], 10/2021 [152]
	VIB4920 [153,154,155,156,157]	CD40L binding protein lacking Fc domain	SS [153], kidney transplant [154], RA [155,156]	I/II	4/2022 [153], 8/2021 [154], 7/2021 [155], 8/2018 [156], 5/2016 [157]
	Letolizumab [158,159]	Fc-silent anti-CD40L dAb	GVHD [158], ITP [159]	I/II	1/2024 [158], 1/2018 [159]

Abbreviations: mAb: monoclonal antibody; dAb: domain antibody; RA: rheumatoid arthritis; ALS: amyotrophic lateral sclerosis; T1DM: type 1 diabetes mellitus; SCC: squamous cell carcinoma; B-CLL: B-cell chronic lymphocytic leukemia; LN: lupus nephritis; CD: Crohn’s disease; MS: multiple sclerosis; ITP immune thrombocytopenia; SS: Sjogren’s syndrome; MG: myasthenia gravis; PBC: primary biliary cirrhosis; NSCLC: non-small cell lung cancer; RCC: renal cell carcinoma; CRC: colorectal cancer; GVHD: graft-versus-host disease; GM-CSF: granulocyte-macrophage colony-stimulating factor; MDS: myelodysplastic syndrome; PDAC: pancreatic ductal adenocarcinoma; FAP-α: fibroblast activation protein-α. Table adapted from Karnell et al. [7]. Red font emphasizes neurological disease for which the treatment is under investigation.

## 4. Therapies Targeting CD40 and CD40L

We define two therapeutic approaches used to combat neurological disease via antagonism or agonism of the CD40–CD40L interaction (Table 1):

(1) Attenuating CD40-mediated neuroinflammation: CD40–CD40L signaling potentiates neuroinflammatory damage in the CNS. The majority of CD40 therapies used in the treatment of autoimmune and neuroinflammatory disorders such as MG, MS, and ALS, exist in the form of antagonistic monoclonal antibodies against CD40 or CD40L and are administered either as a single agent or in combination with other antibodies, chemotherapeutic agents, and/or corticosteroids (Table 1).
  Using this treatment strategy, CD40 antagonists have the potential not only to limit edema, demyelination, BBB permeability, and neural tissue damage, but also to limit the disease-specific mechanisms that CD40 activation typically exacerbates; for example, the dysregulation of amyloid precursor protein (APP) processing and tau phosphorylation that contributes to the formation of neurofibrillary tangles and β-amyloid plaques in AD (Section 3.2).
(2) Employing CD40-mediated recruitment of inflammatory cells to enhance tumor lysis: Motivations for targeting the CD40 axis in cancer treatment include (1) CD40 ligation initiates antigen-specific activation of B and T cells (2) the CD40 axis bridges innate and adaptive immunity as it activates natural killer cells for tumor killing and (3) CD40 expression by antigen presenting cells such as macrophages enhances their antigen presentation and co-stimulatory capacity, allowing for activation of cytotoxic T cells even without CD4+ helper T-cell signaling [160].
  CD40-based therapies tested in in vivo tumor models include recombinant CD40L molecules, intratumor adenoviral vectors which lead to CD40L expression, and agonistic monoclonal CD40 antibodies [160]. CD40 ligation on the surface of neoplastic cells resulted in direct cytotoxic effects, even in the absence of immune accessory cells [161,162,163]. CD40 agonism and resulting tumor cell death was shown to be synergic with chemotherapy in murine models: when combined with gemcitabine and administered to mice with established implanted tumors, most mice were cured and resistant to tumor rechallenge [164].
  Regarding the safety of agonistic CD40 antibodies, clinical trials have noted that adverse events such as cytokine storm, hepatotoxicity, and thromboembolic events were transient and clinically manageable [160]. Trials are underway for the treatment of solid and hematological malignancies both within and outside of the CNS. CD40-agonistic immunotherapies under investigation for the treatment of brain tumors include Sotigalimab and 2141-V11, with expected completion by the end of 2022 and 2025, respectively (Table 1).

## 5. Conclusions

CD40–CD40L signaling leads to a pro-inflammatory microenvironment—a key physiologic response to cellular infection, cancer, and injury. However, this can progress to increased permeability of the BBB, edema, the formation of microthrombi, and subsequent CNS damage. We have reviewed the role of CD40–CD40L in neurological diseases of non-infectious etiology and distinguished two general classes of CD40–CD40L therapies used to combat neurological disease: those that inhibit CD40–CD40L mediated neuroinflammation to attenuate the immune response and those that upregulate CD40 signaling for tumor cell lysis. Both cases present the opportunity to expand and repurpose current CD40 immunotherapies in future translational research as new therapeutic avenues to treat neurological disease.

## Data Availability

Data that support the findings of this review are available by searching the following MeSH terms in PubMed: CD40, Neurological Disease, Glioblastoma.
